# Association between white matter hyperintensities and altered cerebral blood flow in maintenance hemodialysis patients: a longitudinal study

**DOI:** 10.1186/s12882-024-03468-3

**Published:** 2024-01-24

**Authors:** Mingan Li, Wenbo Yang, Lijun Song, Zhenghan Yang, Zhenchang Wang, Junfang Xian, Hao Wang

**Affiliations:** 1 Department of Radiology, Beijing Friendship Hospital, Capital Medical University, No. 95 Yong An Road, Xicheng District, Beijing, 100050 China; 2 Department of Radiology, Beijing Tongren Hospital, Capital Medical University, No.1 of Dongjiaominxiang Street, Dongcheng District, Beijing, 100730 China

**Keywords:** Hemodialysis, White matter hyperintensity, Cerebral blood flow, Arterial spin labeling

## Abstract

**Objectives:**

To explore changes in cerebral blood flow (CBF) and white matter in hemodialysis patients.

**Methods:**

Thirty-three hemodialysis patients who underwent two brain MRI at an interval of three years and 33 age- and sex-matched healthy controls (HC) underwent structural and arterial spin-labeling MRI examinations. Intergroup differences in CBF in the gray matter, white matter, and whole matter, and regional white matter hyperintensities (WMH) were analyzed. Based on the changes in CBF between the baseline and follow-up groups, the hemodialysis patients were divided into two subgroups: an increased CBF group and a decreased CBF group. Differences in CBF and WMH between the subgroups and HC were analyzed.

**Results:**

Patients undergoing hemodialysis exhibited increased cerebral watershed (CW) WMH, deep WMH, and periventricular WMH (*P* < 0.01). The CBF of patients with decreased CBF was higher than that of HC at baseline (,*P* < 0.01) and lower than that of HC at follow-up (*P* < 0.01). Compared with the increased CBF group, obvious development of deep WMH was found in the decreased CBF group for the gray matter, white matter, and whole matter (*P* < 0.01).

**Conclusions:**

WMH in hemodialysis patients were distributed in the deep white matter, periventricular white matter and CW, and progressed with the extension of hemodialysis duration. CBF in hemodialysis patients could manifest as both increased and decreased, and WMH in patients with decreased CBF developed severely with prolongation of hemodialysis duration.

**Advances in knowledge:**

These findings provide a basis for exploring neuropathological changes of hemodialysis patients.

## Introduction

End-stage renal disease (ESRD) [1] is defined as chronic renal disease (CKD) with a glomerular filtration rate (GFR) less than 15 ml/min/1.73 m^2^. Hemodialysis is the most regulated replacement therapy performed in clinical practice for patients with ESRD [2]. However, hemodynamic fluctuations during hemodialysis therapy may result in brain damage, such as enlarged perivascular space (PVS) [3] and white matter hyperintensity (WMH) [4]. Severe WMH is known to be an important factor in the progression of diseases such as stroke, cerebral small vessel disease (CSVD), cognitive decline, and Alzheimer’s disease, which can seriously reduce the quality of life of patients and increase the economic burden [5–7]. Therefore, there is an urgent need to explore WMH in patients with ESRD.

Hemodialysis patients had more WMH compared to the healthy population in the community, and WMH was positively correlated with cognition decline [8, 9]. The WMH penumbra represents the brain white matter lesions around the WMH that appear as normal white matter on conventional MRI and would progress to WMH in the future, which was proposed in a community-based population study on the progression of WMH [10]. The WMH penumbra includes the structural penumbra and cerebral blood flow (CBF) penumbra. The structural penumbra of the WMH, defined by diffusion tensor imaging (DTI) and FLAIR, was the white matter area with microstructural damage on DTI extending approximately 2–9 mm, which was normal on FLAIR around the WMH. In addition, the CBF penumbra of WMH, defined by arterial spin labeling (ASL) and FLAIR, was a white matter area with abnormal CBF extending approximately 12–14 mm, which appeared normal on FLAIR around the WMH. Therefore, we concluded that the destruction of white matter integrity and CBF changes occurred before the appearance of WMH. The CBF penumbra of WMH covered more than the structural penumbra, suggesting that CBF changes might precede the destruction of white matter integrity [10–12]. However, a correlation between WMH and CBF in patients undergoing maintenance hemodialysis has not yet been reported.

A mechanism of altered cerebral blood flow perfusion in patients undergoing hemodialysis has been proposed [13]. In hemodialysis patients with ESRD, anemia results in hypertension, while a decrease in blood viscosity and oxygen-carrying capacity further increases CBF through the mechanism of brain self-regulation [13–15]. CBF can decrease by about 7–22% during hemodialysis [16, 17]. Hypoxic perfusion and alternate changes in CBF between hemodialysis and rest lead to the damage of brain tissue [16, 18], especially cerebrovascular endothelial dysfunction and cerebral perfusion regulation mechanisms, which further leads to the destruction of other structures of brain tissue [19]. ASL can be used to measure CBF, which reflects the activity of brain tissue [20]. Therefore, ASL can be used as an important means to examine the relationship between WMH and CBF.

In this study, we aimed to (1) confirm the characteristics of WMH in hemodialysis patients in terms of space and time using high-resolution T_1_ weighted, T_2_ weighted, and FLAIR images, (2) evaluate the regulation of CBF alteration by ASL, and (3) prove the hypothesis that altered CBF affects the progression of WMH.

## Materials and methods

### Participants

**Fig. 1:**
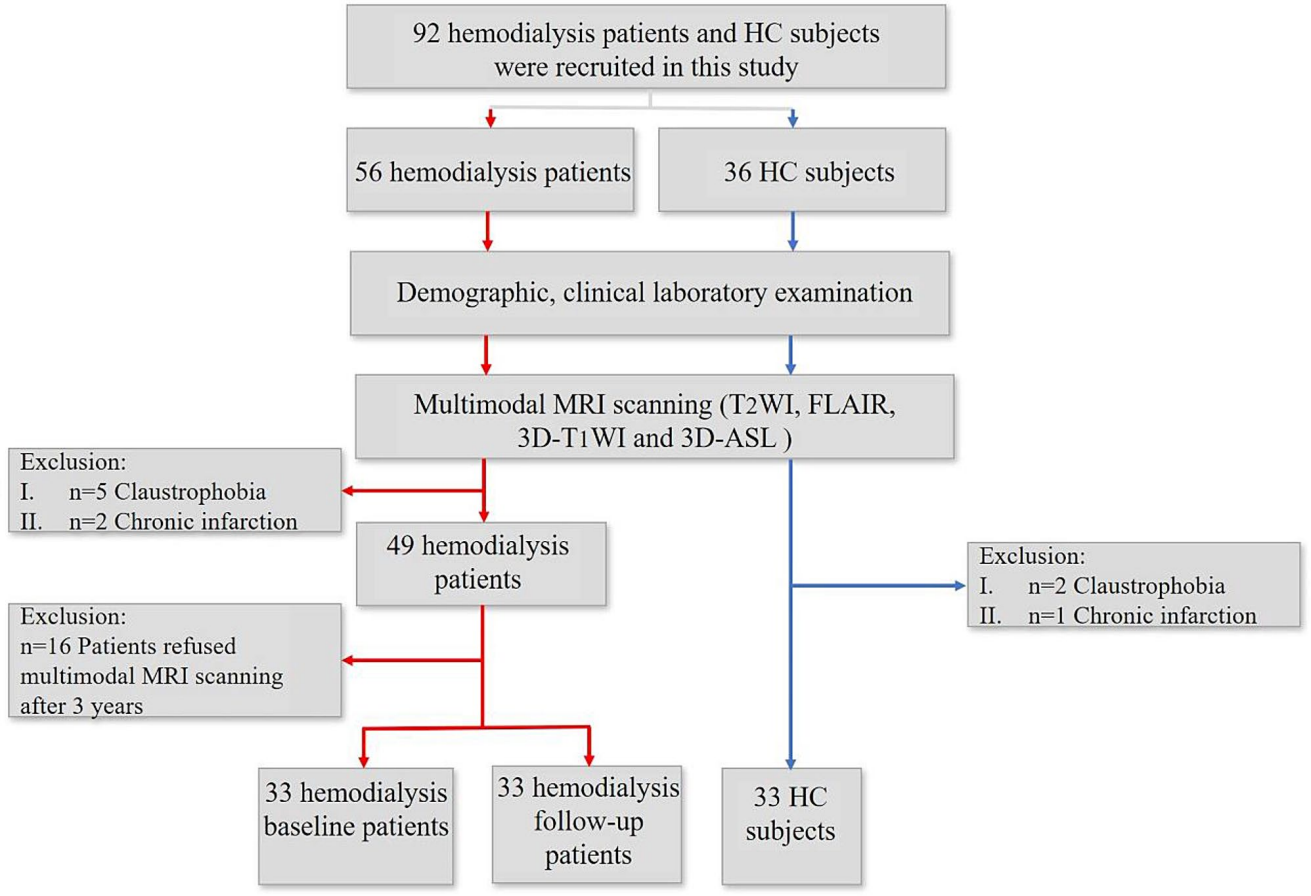
Flowchart of the enrollment of subjects. HC = healthy control; ASL = arterial spin labeling

This study was approved by the Medical Research Ethics Committee of Beijing Friendship Hospital and informed consent was obtained from each participant included in the study. A total of 56 patients on hemodialysis were recruited in hemodialysis center of Beijing Friendship Hospital between August 2018 and March 2019. Hemodialysis was performed using high-flux polysulfone, polyethersulfone, or polymethylmethacrylate membrane dialyzers with a membrane area of 1.5–1.8 m^2^, blood flow of 220–300 mL/min, and ultrapure dialysis fluid (bacteria < 0.1 CFU/mL and endotoxin < 0.03 IU/mL). The composition of the dialysis fluid was: 35 mmol/L bicarbonate, 3.7 mmol/L acetate, 138 mmol/L sodium,1.75 mmol/L calcium, 0.5 mmol/L magnesium, 3.0 mmol/L potassium, 110 mmol/L chloride. The dialysate flow was 500 mL/min, and dialysate temperature was 36.5 °C. Anticoagulation was achieved by heparin or low-molecular-weight heparin. The Kt/V of urea (Kt/V) was ≥ 1.2, and urine output was < 100 mL/day. The inclusion criteria were (I) the age of all participants was > 20 years old, (II) right-handed, (III) MRI scanning was accepted by all participants and (IV) duration of hemodialysis was > 6 months. The exclusion criteria were (I) failure to complete the MRI scans, (II) image quality for analysis, (III) other brain diseases affecting image changes, including chronic infarction, trauma, tumors, and some neurodegenerative diseases, (IV) presence of diabetic nephropathy or diabetes, (V) history of peritoneal dialysis or kidney transplantation. According to the inclusion and exclusion criteria, 33 patients underwent a baseline examination and second examination at a follow-up of approximately 3 years. All patients underwent 4-hour hemodialysis treatments three times per week as a standard. Thirty-three age- and sex-matched healthy controls (HC) were recruited from local communities in our city (Fig. 1). In 33 hemodialysis patients, 3 arteriovenous fistula with autologous blood vessels and 30 arteriovenous fistula with artificial blood vessels were performed in the left upper limb.

The hemodialysis follow-up patients showed changes in CBF compared to the hemodialysis patients at baseline. Based on the changes in CBF between baseline and follow-up, hemodialysis patients were divided into an increased CBF group and a decreased CBF group. Patients in increased CBF group had increased CBF at follow-up compared to baseline, whereas patients in decreased CBF group had decreased CBF at follow-up compared to baseline. Fifteen of the 33 HC were selected as HC subgroup, whose age and gender were match with the increased and decreased subgroups.

### WMH assessment

**Fig. 2:**
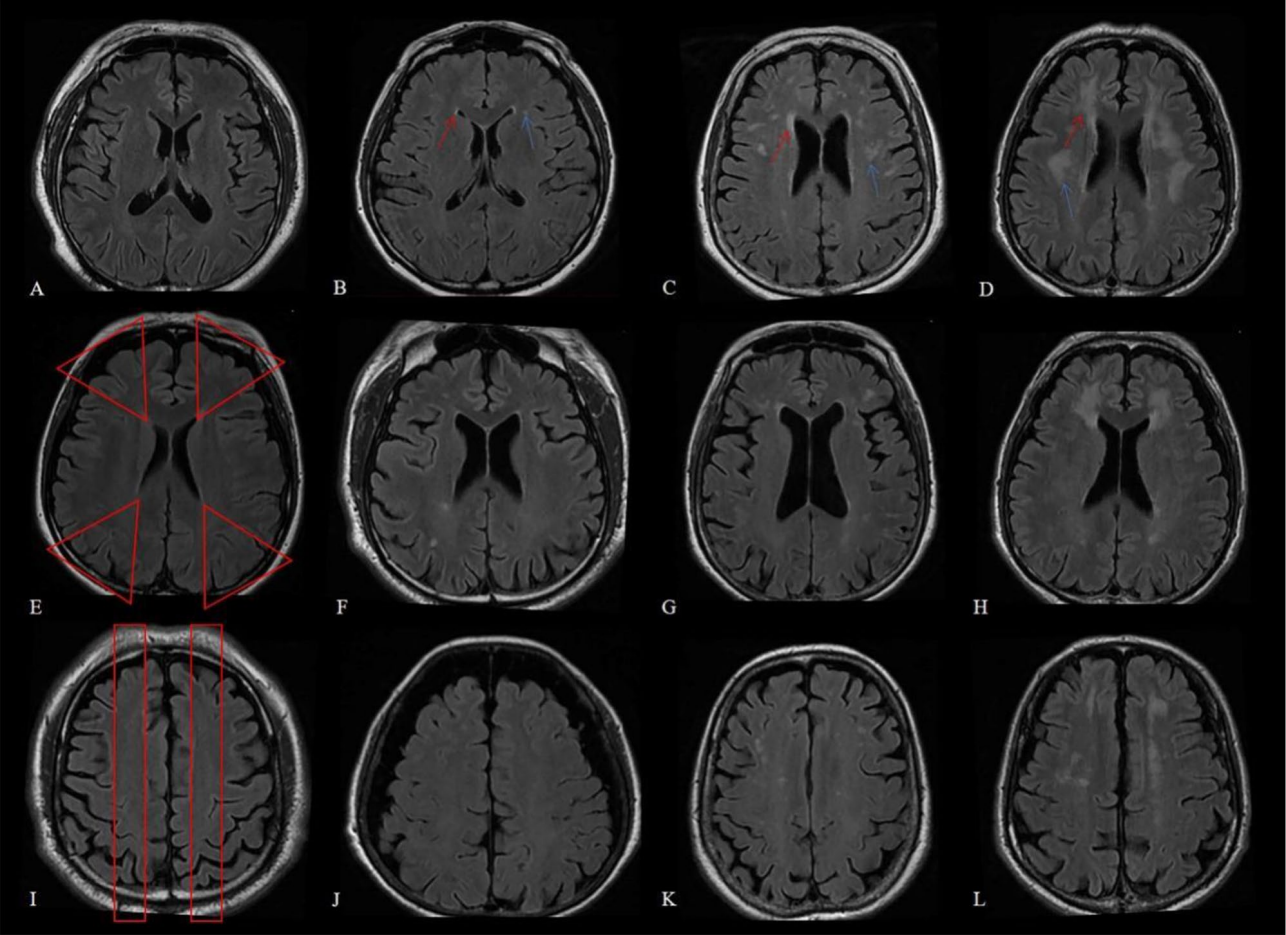
Examples of different categories of WMH scoring from 0 to 3 in the periventricular and deep white matter areas and CW area. FLAIR (**A**-**D**) showed the WMH in the periventricular (red arrow) and deep white matter areas (blue arrow), and from left to right, the scores went from 0 to 3 by the Fazekas visual rating scale [21, 22]. FLAIR (**E**-**H**) showed the WMH in the lower-level CW area (marked red), and from left to right, the scores went from 0 to 3. FLAIR (**I**-**L**) showed the WMH in the up-level CW area (marked red), and from left to right, the scores went from 0 to 3. WMH = white matter hyperintensities; CW = cerebral watershed

WMH is characterized by bilateral, mostly symmetrical hyperintensities on T_2_ weighted images, which appear as isointense or hypointense on T_1_ weighted images (but not as hypointense as the CSF). WMH are classified as hyperintensities of the periventricular and deep white matter areas, which can be semi-quantitatively evaluated using the Fazekas visual rating scale [21, 22] (Fig. 2A-D). WMH in cerebral watershed (CW) areas can also be graded (absence = 0; punctate foci = 1; beginning confluence of foci = 2; large confluent areas = 3), similar to WMH in deep white matter (Fig. 2E-L). The CW area can be divided into upper- and lower-level areas. The sum of the WMH scores in the upper- and lower-level CW areas is the WMH score for the total CW area. All data were analyzed by two experienced neuroradiologists who were blinded to the clinical data.

### MRI data acquisition

Brain MR scans of the participants were performed on a 3.0-Tesla MR system with an eight-channel phased-array coil (Discovery MR750W, General Electric, Milwaukee, Wisconsin, USA). All food or drinks containing caffeine were prohibited before MRI scanning. During scanning, foam padding was used to reduce head motion, and earplugs were provided to minimize scanner noise. The 3D pseudo-continuous arterial spin-labeling (3D-pCASL) sequence was used for brain perfusion. The major parameters for the imaging areas followede 3D-pCASL (post label delay:2025ms; repetition time (TR):4844 ms; echo time (TE):10.5 ms; matrix:128 × 128; field of view (FOV):240 × 240 mm; number of excitations:3; spiral-in readout using FSE imaging, 512 sample points with eight arms, and several label and control images:72; number of axial slices:36, slice thickness:4 mm). The overall acquisition time of 3D-pCASL performance across whole-brain coverage was 4 min 41 s. A T_1_ weighted structural image (TR:8.8 ms, TE:3.5 ms; inversion time (TI):450 ms; number of sagittal slices:196; slice thickness:1 mm (no gap); matrix:256 × 256; FOV:240 × 240 mm; flip angle:15°). T_2_WI (TR,7299 ms; TE,169 ms; number of axial slices,20; slice thickness,6 mm; matrix,256 × 256; FOV,240 × 240 mm; flip angle,15°). FLAIR images (TR,8525 ms; TE,140 ms; flip angle,111°; matrix,256 × 256). During the scans, all participants were asked to relax, keep their eyes closed, think of nothing in particular, and avoid falling asleep.

### Cerebral blood flow (CBF) calculation

CBF calculations were performed using SPM 12 software (Statistical Parametric Mapping, Institute of Neurology, London, UK) on the MATLAB platform (R2018b; MathWorks), as described in our previous study [3]. First, a single-compartment model was used to estimate the ASL difference image after subtracting the labeled image from the control image. CBF maps were estimated by averaging three ASL difference images combined with proton density-weighted reference images [23–24]. Second, a positron emission tomography (PET) perfusion template in the Montreal Neurological Institute (MNI) space was coregistered to the CBF images of the 33 HC using nonlinear transformation in SPM 12. The mean coregistered CBF map of the 33 HC was used as a standard CBF template. Third, the CBF maps of all participants were coregistered to the standard CBF template of the MNI and resampled to a voxel size of 2 × 2 × 2 mm. Finally, an 8-mm full-width at half-maximum (FWHM) Gaussian kernel was used to smooth each co-registered CBF image. Finally, we obtained the CBF of the gray matter, white matter, and whole matter.

Decreasing hemoglobin level is common in hemodialysis patients. Relaxation time (T_1_) of blood (T_1_B) varies with age and hematocrit or hemoglobin levels [25]. T_1_B could modified CBF quantification results significantly. To get rigorous CBF quantification, a single compartment model was used for CBF quantification. The hemoglobin levels of participants and the previously proposed general model were used to estimate the T_1_B in this study [3]. The hemoglobin levels of hemodialysis patients were derived from blood samples obtained during standard hemodialysis treatments; an age- and sex-dependent hemoglobin level was applied for HC. T_1_B was estimated by using a fixed normal arterial blood oxygen saturation level of 0.97 for all participants.

### Statistical analysis

Statistical analyses were performed using SPSS software (version 23.0, Chicago, IL, USA). Differences in sex between patients undergoing hemodialysis and HC were analyzed using the chi-square test. Differences in age, gray matter CBF, white matter CBF, and whole matter CBF among HC, hemodialysis baseline patients, and hemodialysis follow-up patients were analyzed using the ANOVA. A paired-samples *t*-test was conducted to evaluate the differences in CBF between baseline and follow-up of patients on hemodialysis, and an independent-sample *t*-test was used to study the differences between baseline hemodialysis patients and HC. The differences in WMH in the periventricular, deep white matter, and CW areas among HC, baseline hemodialysis patients, and hemodialysis follow-up patients were assessed using the Kruskal–Wallis test. The Wilcoxon signed-rank test was conducted to evaluate the differences in WMH scores between baseline and follow-up patients on hemodialysis, and the Mann–Whitney *U* test was used for baseline hemodialysis patients and HC.

Increased CBF, decreased CBF, and HC groups were analyzed using Tukey’s test. For multiple comparisons, tests were corrected using the Bonferroni method (*P* < 0.05/3 = 0.017). The chi-square test for WMH in the periventricular area, deep white matter area, and CW area was performed between the increased and decreased CBF groups of hemodialysis patients.

## Results

### Demographics and hemodialysis duration

There were no significant differences in age and sex among the baseline hemodialysis patients, hemodialysis follow-up patients, and HC (*P* = 0.121 and 1.000, respectively). The demographics of the study population are presented in Table 1.

**Table 1:** Demographic, WMH scores and CBF of the study participants

	Hemodialysis baseline group (*n* = 33)	Hemodialysis follow-up group (*n* = 33)	HC group (*n* = 33)	P
Age (years)	53.7 ± 10.1	56.7 ± 10.1	51.8 ± 8.8	0.121
Sex (male/female)	20/13	20/13	20/13	1.000
Hemodialysis duration (months)	84.4 ± 68.6	120.2 ± 68.9	NA	NA
dWMH score	1(0,2)	1(1,2)	0(0,1)	0.000
pWMH score	1(1,2)	1(1,2)	1(1,1)	0.002
Up-level CW-WMH score	1(0,1)	1(0,2)	0(0,1)	0.001
Lower-level CW-WMH score	1(0,2)	1(0,2)	0(0,1)	0.000
Total CW-WMH score	1(0,3)	2(1,4)	0(0,1.5)	0.000
Grey matter CBF (ml/100 g/min)	44.638 ± 9.161	44.784 ± 7.946	47.723 ± 8.433	0.258
White matter CBF (ml/100 g/min)	39.423 ± 7.678	40.575 ± 7.148	40.252 ± 6.156	0.790
Whole matter CBF (ml/100 g/min)	41.997 ± 8.565	42.719 ± 7.320	44.611 ± 7.655	0.382
Pre-dialysis MAP (mmHg)	96.5 ± 10.1	98.1 ± 12.1	NA	0.076
Post-dialysis MAP (mmHg)	99.3 ± 11.9	98.9 ± 11.1	NA	0.632
Hb (g/L)	116.5 ± 11.6	117.6 ± 11.6	NA	0.754
UFR (mL/h)	679.4 ± 164.5	682.4 ± 156.7	NA	0.456
ALB (g/L)	38.0 ± 2.7	38.4 ± 2.8	NA	0.162
hs-CRP (mg/L)	3.2 (1.4–8.6)	2.9 (1.4–8.9)	NA	0.487

Data are presented as the mean ± standard deviation, median (upper and lower quartiles)

WMH = white matter hyperintensities; CBF = cerebral blood flow; HC = healthy controls; NA = not applicable; dWMH = deep white matter hyperintensities; pWMH = periventricular white matter hyperintensities; CW-WMH = cerebral watershed white matter hyperintensities; MAP = Mean arterial pressure; UFR = ultrafiltration rate; ALB = albumin hs-CRP = hypersensitive C-reactive protein

### The differences of WMH between HC and hemodialysis baseline patients

Baseline hemodialysis patients had higher WMH scores than the HC group in the deep white matter area, up-level CW area, lower-level CW area, and total CW area (*P* = 0.005, 0.008, 0.005, and 0.002, respectively). There was no significant difference in periventricular WMH scores between baseline hemodialysis patients and HC (*P* = 0.059) (Table 1).

### The changes of WMH between hemodialysis baseline patients and hemodialysis follow-up patients

The hemodialysis follow-up patients had higher WMH scores than the baseline hemodialysis patients in the deep white matter, periventricular white matter, upper CW, lower CW, and total CW areas (*P* = 0.013, 0.008, 0.004, 0.029, and 0.002, respectively) (Table 1).

### Changes of CBF in HC, hemodialysis baseline patients and hemodialysis follow-up patients

**Fig. 3:**
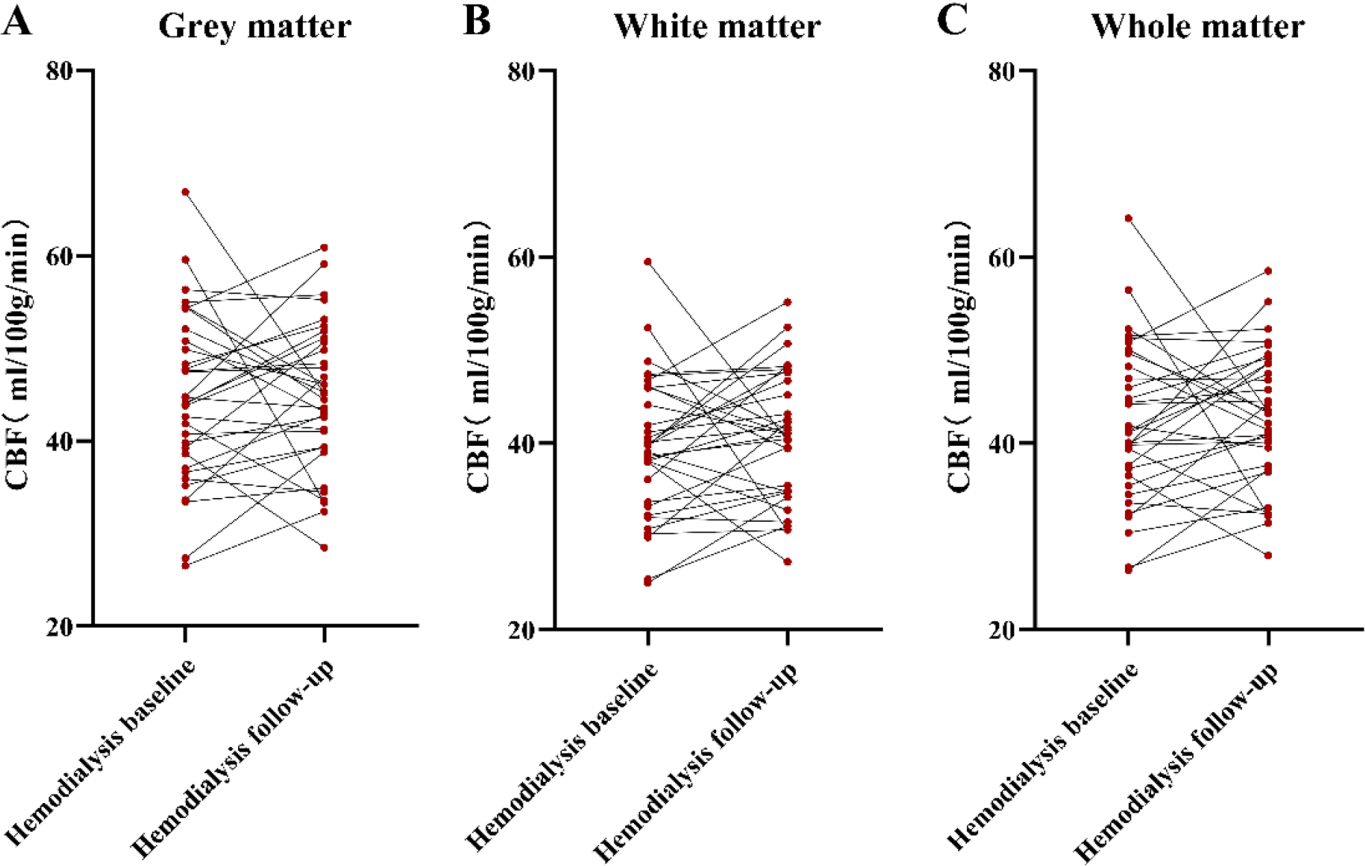
Comparison of CBF between the baseline and follow-up hemodialysis patients No difference for CBF between the baseline and follow-up hemodialysis patients was found in the grey matter (**A**), white matter (**B**) and whole matter (**C**). (*P* > 0.05). CBF = cerebral blood flow

The CBF of gray matter, white matter, and whole matter showed no significant differences among HC, hemodialysis follow-up patients, and hemodialysis baseline patients (Table 1; Fig. 3A-C).

### Changes of CBF and WMH scores in increased CBF group, decreased CBF group and HC group for subgroup

**Fig. 4:**
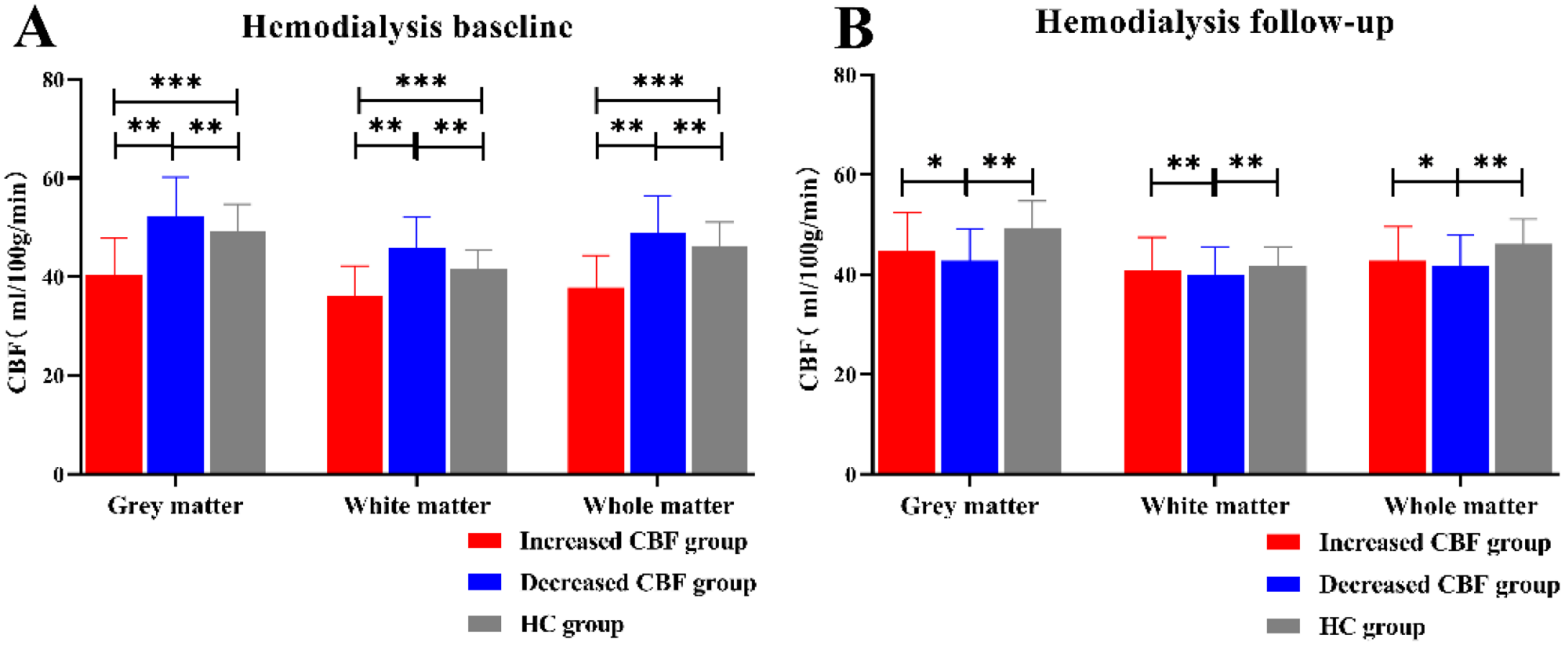
The measures of CBF among the gray matter, white matter, and whole matter in the hemodialysis baseline group and hemodialysis follow up group. ***Significant group differences with Tukey’s test correction, *P* < 0.001, ** Significant group differences with Tukey’s test correction, *P* < 0.01, * Significant group differences with Tukey’s test correction, *P* < 0.05. (**A**) CBF of decreased CBF group in the grey matter, white matter and whole matter was higher than increased CBF group (*P* < 0.01) and higher than HC group (*P* < 0.01) in hemodialysis baseline. (**B**) CBF of decreased CBF group in the grey matter, white matter and whole matter was lower than increased CBF group (*P* < 0.01) and lower than HC group (*P* < 0.01) in hemodialysis follow-up. CBF = cerebral blood flow; HC = healthy control; GM = grey matter; WM = white matter

The CBF values in the gray matter, white matter, and whole matter in the decreased CBF group were higher than those in the HC group at baseline (*P* < 0.01). However, the CBF in the decreased-CBF group in the gray matter, white matter, and whole matter was lower than that in the HC group at follow-up (*P* < 0.01). (Fig. 4A,B)

The WMH scores in the deep white matter region progressed more in the decreased CBF group than in the increased CBF group (Table 2). However, no obvious progression was observed in the periventricular white matter, upper CW, lower CW, or total CW areas (Table 2).

**Table 2:** The variations of WMH scores from baseline to follow-up for hemodialysis CBF increased and decreased patients

	Grey matter CBF			White matter CBF			Whole matter CBF		
	increased	decreased	P	increased	decreased	P	increased	decreased	P
dWMH variation (+/-)	1/14	8/10	0.015	1/15	8/9	0.009	1/15	8/9	0.009
pWMH variation (+/-)	3/12	4/14	0.876	3/13	4/13	0.737	3/13	4/13	0.737
Up-level CW-WMH variation (+/-)	3/12	6/12	0.392	3/13	6/11	0.286	3/13	6/11	0.286
Lower-level CW-WMH variation (+/-)	2/13	7/11	0.101	2/14	7/10	0.065	2/14	7/10	0.065
total CW-WMH variation (+/-)	4/11	9/9	0.172	4/12	9/8	0.101	4/12	9/8	0.101

WMH = white matter hyperintensities; CBF = cerebral blood flow; dWMH = deep white matter hyperintensities; pWMH = periventricular white matter hyperintensities; CW-WMH = cerebral watershed white matter hyperintensities

## Discussion

In this study, the spatiotemporal distribution of WMH and the characteristics of CBF changes in hemodialysis patients were revealed. Compared to the HC group, the hemodialysis baseline and follow-up patients exhibited increased scores for cerebral watershed white matter hyperintensity (CW-WMH), deep WMH, and periventricular WMH, which progressed with the extension of the duration of hemodialysis. Among HC, hemodialysis baseline, and follow-up patients, the CBF of gray matter, white matter, and whole matter showed no obvious differences. However, after the hemodialysis patients were divided into an increased CBF group and a decreased CBF group according to the changes in CBF between the baseline and follow-up groups, we found that the CBF of the patients with decreased CBF was higher than that of HC at baseline and lower than that of HC at follow-up. Moreover, we conducted a comparative analysis of WMH between the increased CBF group and the decreased CBF group and found that patients in the decreased CBF group had a more obvious development of deep WMH than those in the CBF increase group. Although there was no statistically significant difference in the CW-WMH scores, the p-values ranged from 0.1 to 0.4, suggesting a tendency for CW-WMH to increase with decreasing CBF.

The CW, deep white matter, and periventricular white matter areas may be prone areas for WMH to appear in hemodialysis patients. Our study indicated that hemodialysis patients had more WMH than the healthy population in the community, which is consistent with previous research [4, 8]. Furthermore, significant progression of WMH in the CW, deep white matter, and periventricular white matter areas was observed in hemodialysis patients after approximately three years. In our previous research, we found that the number of enlarged perivascular space in the centrum semiovale and cerebral watershed had a significant positive correlation with hemodialysis duration in the hemodialysis patients [3]. Interestingly, we found similar results in the WMH in the hemodialysis baseline patients. Thus, we proposed for the first time that in hemodialysis patients, the CW area was more likely to develop WMH, which progressed with hemodialysis duration. The spatiotemporal characteristics of WMH in hemodialysis patients may provide an objective basis for imaging diagnosis and follow-up of brain impairment in patients with renal disease in the future.

Previous studies have reported conflicting conclusions regarding the characteristics of CBF changes in patients undergoing hemodialysis. Some studies have suggested that CBF in hemodialysis patients is increased in the whole brain compared to that in the normal population [13, 14]. However, Li [25] found that after approximately 6 months of hemodialysis, CBF declined substantially in hemodialysis patients and became comparable to that in healthy controls. Our study showed a similar conclusion in that there were no significant differences in white matter CBF, gray matter CBF, and whole matter CBF between hemodialysis patients and HC. Anemia due to renal failure can cause higher blood viscosity and lower oxygen delivery, resulting in excessive cerebral blood perfusion. The effect leading to increased CBF was associated with hematocrit levels. Hemodialysis treatment reduces blood pressure, intracranial pressure, and cerebral perfusion pressure, and ultimately leads to a decrease in CBF [13, 20, 26]. Therefore, CBF in hemodialysis patients has a complex change regulation.

To further explore the changes in CBF in hemodialysis patients, we divided them into an increased CBF group and a decreased CBF group according to the changes in CBF at an interval of three years. The CBF in the decreased CBF group decreased from higher than that of HC at baseline examination to lower than that of HC at follow-up examination. This may be due to the aforementioned dynamic balance between the mechanisms of CBF regulation and compensatory mechanisms in hemodialysis patients.

In addition, regression to the mean CBF, according to CBF regulation, may be another possible reason. Further longitudinal mechanistic studies are required to confirm these findings.

In addition, we found that the WMH of the CW, deep white matter, and periventricular white matter areas in the decreased CBF group progressed significantly more than those in the increased CBF group after three years. Previous studies have found that WMH become increasingly obvious with the prolongation of duration of hemodialysis. However, the relationship between the change in WMH and change in CBF has not been clearly explored. Some studies have focused on the progression of WMH in the community population. WMH penumbras represent milder WM injuries than WMH and progress to WMH in the future. CBF WMH penumbrae cover approximately 12–14 mm surrounding the WMH, suggesting that a decrease in CBF precedes WMH [11, 12]. This is in agreement with the results of our study on hemodialysis patients. CBF fluctuations in patients undergoing hemodialysis play a key role in brain impairment in patients with renal disease. Although the increased CBF partially compensated for the oxygen supply in the brain tissue, the decreased oxygen-carrying capacity of the blood made the oxygen supply to the brain insufficient, eventually leading to brain tissue damage [13]. Moreover, CBF decreases during hemodialysis, which further worsens the oxygenation of brain tissue [13, 16–17]. Because there are fewer capillaries in the white matter than in the gray matter, brain injury in the white matter is more obvious, such as demyelination of the white matter and neuronal necrosis, which present as WMH in several types of researches [3, 8, 27]. Therefore, a decrease in CBF in patients undergoing hemodialysis may be an important factor in WMH.

Our study had several limitations. First, the sample size was relatively small. To discuss and analyze the spatiotemporal characteristics of WMH and CBF in patients on hemodialysis, larger sample sizes should be collected. Second, the experimental design can only explore the correlation between WMH and CBF but cannot clarify the causal relationship.

## Conclusion

In conclusion, we demonstrated that WMH in hemodialysis patients were distributed in the deep white matter, periventricular white matter, and CW, and it aggravated with time. CBF in hemodialysis patients can be manifested as both increased and decreased, and the progression of WMH in patients with decreased CBF was more obvious with prolonged duration of hemodialysis.

## Data Availability

The data that supports the findings of this study is not publicly available since restrictions from the Beijing Friendship Hospital, Capital Medical University apply to the availability of these data. However, the data are available from the authors (Hao Wang and Zhenchang Wang) upon reasonable request and with permission of the Beijing Friendship Hospital, Capital Medical University.
